# 

**Published:** 1992-08

**Authors:** 


W1 WE403
V.107 NO.2 1992
C.01--------SEQ: SR0068788
TI: WEST OF ENGLAND MEDICAL
JOURNAL

### WEST OF ENGLAND MEDICAL JOURNAL

**Volume 107(ii)** **August 1992**

NATIONAL LIBRARY
FEB 8 1993
OF MEDICINE

**Tuberculosis — The Next Plague?**
**The Great Pretender**
**A Peptide Family Being Re-United: The Angiotensins Coming in from the Cold**
**Crossword**
**The 100th Day Case Disc**
**The Mobile Hospitals of the British Red Cross and the Order of St. John and their Role in the Rotterdam Typhoid Epidemic 1945**
**Factors which Predispose Elderly Patients to Develop Peptic Ulcer Disease**
**Give us back our 11 days**
**Rheumatology and its inception in the South West**
**Sir William Osler, Oxford and "The Open Arms"**
**South West Orthopaedic Club**
**South West Vascular Surgeons**
**South West Surgical Club**
**South West Obstetrical and Gynaecological Society**

FORMERLY BRISTOL MEDICO-CHIRURGICAL JOURNAL

ISSN: 0960 6440 NLM Code B5Q

